# Cardiac electrophysiology in mice: a matter of size

**DOI:** 10.3389/fphys.2012.00345

**Published:** 2012-09-05

**Authors:** Sven Kaese, Sander Verheule

**Affiliations:** ^1^Division of Experimental and Clinical Electrophysiology, Department of Cardiology and Angiology, University Hospital MünsterMünster, Germany; ^2^Department of Physiology, Faculty of Medicine, Maastricht UniversityMaastricht, Netherlands

**Keywords:** arrhythmias, atrial fibrillation, conduction, mouse, scaling, ventricular fibrillation

## Abstract

Over the last decade, mouse models have become a popular instrument for studying cardiac arrhythmias. This review assesses in which respects a mouse heart is a miniature human heart, a suitable model for studying mechanisms of cardiac arrhythmias in humans and in which respects human and murine hearts differ. Section I considers the issue of scaling of mammalian cardiac (electro) physiology to body mass. Then, we summarize differences between mice and humans in cardiac activation (section II) and the currents underlying the action potential in the murine working myocardium (section III). Changes in cardiac electrophysiology in mouse models of heart disease are briefly outlined in section IV, while section V discusses technical considerations pertaining to recording cardiac electrical activity in mice. Finally, section VI offers general considerations on the influence of cardiac size on the mechanisms of tachy-arrhythmias.

## Scaling of cardiac physiology to body mass

Body mass varies between small mammals like the mouse (0.03 kg, see Table 1) to the largest mammal, the blue whale (30,000 kg) by a factor of 10^6^ (Noujaim et al., 2004). Very diverse physiological parameter such as basal metabolic rate, life span, left ventricular ejection time, and ECG time intervals (RR, PR, QRS, and QT) scale with body mass (BM) (West et al., 1997, 1999; Noujaim et al., 2004, 2007; Popovic et al., 2005). The “universal law of allometric scaling” for a parameter **P** is expressed by the allometric equation: **P = aBM**^**b**^ (West et al., 1997, 1999; Noujaim et al., 2007), where ***a*** is a normalization constant and ***b*** represents the scaling exponent. Frequently, ***b*** is a multiple of 1/4. Basal metabolic rate scales as BM^3/4^ (West et al., 1997, 1999) and is thought to be related to the ratio of body surface area to body volume. A larger body surface-to-volume ratio results in faster temperature loss of the body and thus requires a higher basal metabolic rate to maintain body temperature. Mice, with a high surface-to-volume ratio, tend to have a relatively high basal metabolic rate (see Table 1). Adequate blood pressure is required to maintain homeostasis and basal metabolic rate. The mean arterial pressure does not scale with body mass and is thus similar between mice and humans (West et al., 1997; Janssen and Smits, 2002). By contrast, cardiac output (CO, exponent 3/4), cardiac stroke volume (SV, exponent 1), blood volume (exponent 1) and circulation time (exponent 1/4) (West et al., 1997; Janssen and Smits, 2002) scale with BM. In mice, CO amounts to 0.0085–0.027 L/min (Table 1) and thus the murine blood volume of 0.002–0.003 L can circulate around 3–14 times per minute. Humans have a CO of 4–8 L/min and a blood volume of 4.5–5.5 L (Table 1), which circulates circa 1–2 times per minute. The cardiac index (CI) relates (CO = SV^*^HR) to body surface area (CI = CO/body surface area). This hemodynamic parameter represents cardiac performance normalized to body surface area and thus indirectly to basal metabolic rate. The cardiac index is tenfold higher in mice than in humans (Janssen and Smits, 2002), reflecting a higher basal metabolic rate in mice. Normalized SV is similar between mice and humans at around 1 μl/g body weight. Therefore, the difference in HR is mainly responsible for differences in CI.

**Table 1:** **Comparison of mouse and human (cardiac) physiology**.

	**Human**	**References**	**Mouse**	**References**
**GENERAL**				
Body mass (kg)	58–85	de la Grandmaison et al., 2001; Stein et al., 2002; Noujaim et al., 2004; Johnstone et al., 2005; Kasper et al., 2008; Later et al., 2010; Barnes et al., 2012	0.015–0.043	Sheng et al., 1999; Verheule et al., 1999; VanderBrink et al., 2000; Janssen and Smits, 2002; Speakman et al., 2002; Noujaim et al., 2004; Xiao et al., 2004; Muller et al., 2005; Hong et al., 2008; Barwe et al., 2009; Brands et al., 2010; Carlstrom et al., 2010; Gros et al., 2010; Carroll et al., 2011
Lifespan (year)	70–80	Zhang and Zhang, 2009	2–2.5	Speakman et al., 2002; Zhang and Zhang, 2009
Basal metabolic rate (kJ/d)	6279	Johnstone et al., 2005	15.6	Speakman et al., 2002
Basal metabolic rate (O2 consumption L/(kg^*^h)	0.9	Janssen and Smits, 2002	0.8–3	Desai et al., 1997; Janssen and Smits, 2002
**HEART**				
Heart weight (g)	261–366	de la Grandmaison et al., 2001; Cunha et al., 2002; Janssen and Smits, 2002; Later et al., 2010	0.12–0.17	Muller et al., 2005; Hong et al., 2008; Barwe et al., 2009; Carlstrom et al., 2010; Carroll et al., 2011
Heart weight/body weight ratio (kg/kg)	0.004–0.006	de la Grandmaison et al., 2001; Cunha et al., 2002; Later et al., 2010	0.004–0.005	Muller et al., 2005; Hong et al., 2008; Barwe et al., 2009; Carlstrom et al., 2010; Carroll et al., 2011
**HEMODYNAMIC**				
Stroke volume (mL)	50–100	Janssen and Smits, 2002; Meijler et al., 2005; Kasper et al., 2008	0.015–0.05	Janssen and Smits, 2002; Fabritz et al., 2010; Gros et al., 2010; Hyyti et al., 2010; Maslov et al., 2010
Cardiac output (L/min)	4–8	Janssen and Smits, 2002; Mestas and Hughes, 2004; Meijler et al., 2005; Kasper et al., 2008	0.005–0.03	Janssen and Smits, 2002; Mestas and Hughes, 2004; Gros et al., 2010; Maslov et al., 2010
Blood pressure (mean arterial pressure, mmHg)	88–100	Mancia et al., 1983; Janssen and Smits, 2002; Barnes et al., 2012; Damkjaer et al., 2012	73–125	Kass et al., 1998; Sheng et al., 1999; Janssen and Smits, 2002; Brands et al., 2010; Carlstrom et al., 2010
Blood volume (L)	5–6	Janssen and Smits, 2002; Mestas and Hughes, 2004; Kasper et al., 2008	0.002–0.03	Sheng et al., 1999; Janssen and Smits, 2002; Mestas and Hughes, 2004
**CARDIAC EP**				
Heart rate (beats/min)	56–101	Edvardsson et al., 1984; Franz et al., 1987; Janssen and Smits, 2002; Stein et al., 2002; Noujaim et al., 2004; Monnig et al., 2005; Zhang and Zhang, 2009; Barnes et al., 2012; Rich et al., 2012	500–724	Kass et al., 1998; Gehrmann et al., 2000; Janssen and Smits, 2002; Xiao et al., 2004; Fabritz et al., 2010
PR interval (ms)	120–200	Stein et al., 2002; Waldek, 2003; Noujaim et al., 2004; Kasper et al., 2008; Grecu et al., 2009	30–56	Thomas et al., 1998; Jalife et al., 1999; Morley et al., 1999; Vaidya et al., 1999; VanderBrink et al., 1999, 2000; Verheule et al., 1999; Gehrmann et al., 2000; Maguire et al., 2000; Saba et al., 2000; Tamaddon et al., 2000; Wehrens et al., 2000; Li et al., 2004, 2009; Noujaim et al., 2004; Saba et al., 2005; Zhang et al., 2005; Sawaya et al., 2007; Mancarella et al., 2008; Stein et al., 2009
QRS duration (ms)	84–110	Kasper et al., 2008; Grecu et al., 2009; Rich et al., 2012	9–30	Thomas et al., 1998; Jalife et al., 1999; Morley et al., 1999; Vaidya et al., 1999; VanderBrink et al., 1999, 2000; Verheule et al., 1999; Gehrmann et al., 2000; Maguire et al., 2000; Saba et al., 2000; Tamaddon et al., 2000; Wehrens et al., 2000; Schrickel et al., 2002; Alcolea et al., 2004; Xiao et al., 2004; Saba et al., 2005; Sawaya et al., 2007; Schrickel et al., 2007; Stein et al., 2009; Gros et al., 2010
QT (ms)	385	Rich et al., 2012	29–109	Thomas et al., 1998; Jalife et al., 1999; Morley et al., 1999; Vaidya et al., 1999; VanderBrink et al., 1999; Gehrmann et al., 2000; Maguire et al., 2000; Saba et al., 2000; VanderBrink et al., 2000; Wehrens et al., 2000; Schrickel et al., 2002; Li et al., 2004; Xiao et al., 2004; Saba et al., 2005; Schrickel et al., 2007; Fabritz et al., 2010; Gros et al., 2010
QTc (ms)	398–430	Stein et al., 2002; Rich et al., 2012	30–124	Gehrmann et al., 2000; Maguire et al., 2000; Saba et al., 2000; Schrickel et al., 2002, 2007; Li et al., 2004; Zhang et al., 2005; Sawaya et al., 2007; Stein et al., 2009; Gros et al., 2010
Atrial ERP (ms)	172–245	Chiamvimonvat et al., 1998; Schauerte et al., 2001; Dorian et al., 2007; Roberts-Thomson et al., 2009; Stiles et al., 2010	23–71	Thomas et al., 1998; VanderBrink et al., 1999; Verheule et al., 1999; van Veen et al., 2005; Zhang et al., 2005; Sawaya et al., 2007; Schrickel et al., 2007; Li et al., 2009; Odening et al., 2009
Atrial CV (cm/s)	88	Hansson et al., 1998	30–60	Thomas et al., 1998; Verheule et al., 1999; Alcolea et al., 2004; van Veen et al., 2005
AV Wenckebach CL (ms)	329–453	Chiamvimonvat et al., 1998; Schauerte et al., 2001; Stein et al., 2002; Kose et al., 2004; Dorian et al., 2007; Grecu et al., 2009	66–133	Thomas et al., 1998; Hagendorff et al., 1999; VanderBrink et al., 1999; Verheule et al., 1999; Maguire et al., 2000; Saba et al., 2000; VanderBrink et al., 2000; Korte et al., 2002; Schrickel et al., 2002; Saba et al., 2005; Zhang et al., 2005; Sawaya et al., 2007; Schrickel et al., 2007; Li et al., 2009; Blana et al., 2010
Ventricular ERP (ms)	223–257	Edvardsson et al., 1984; Chiamvimonvat et al., 1998; Schauerte et al., 2001; Dorian et al., 2007	33–80	Thomas et al., 1998; Verheule et al., 1999; Kovoor et al., 2001; Korte et al., 2002; Saba et al., 2005; van Veen et al., 2005; Zhang et al., 2005; Sawaya et al., 2007; Schrickel et al., 2007; Li et al., 2009; Stein et al., 2009
Ventricular CV (cm/s)	80	de Bakker et al., 1993	30–60	Thomas et al., 1998; Morley et al., 1999; Alcolea et al., 2004; van Veen et al., 2005

**Figure 1:**
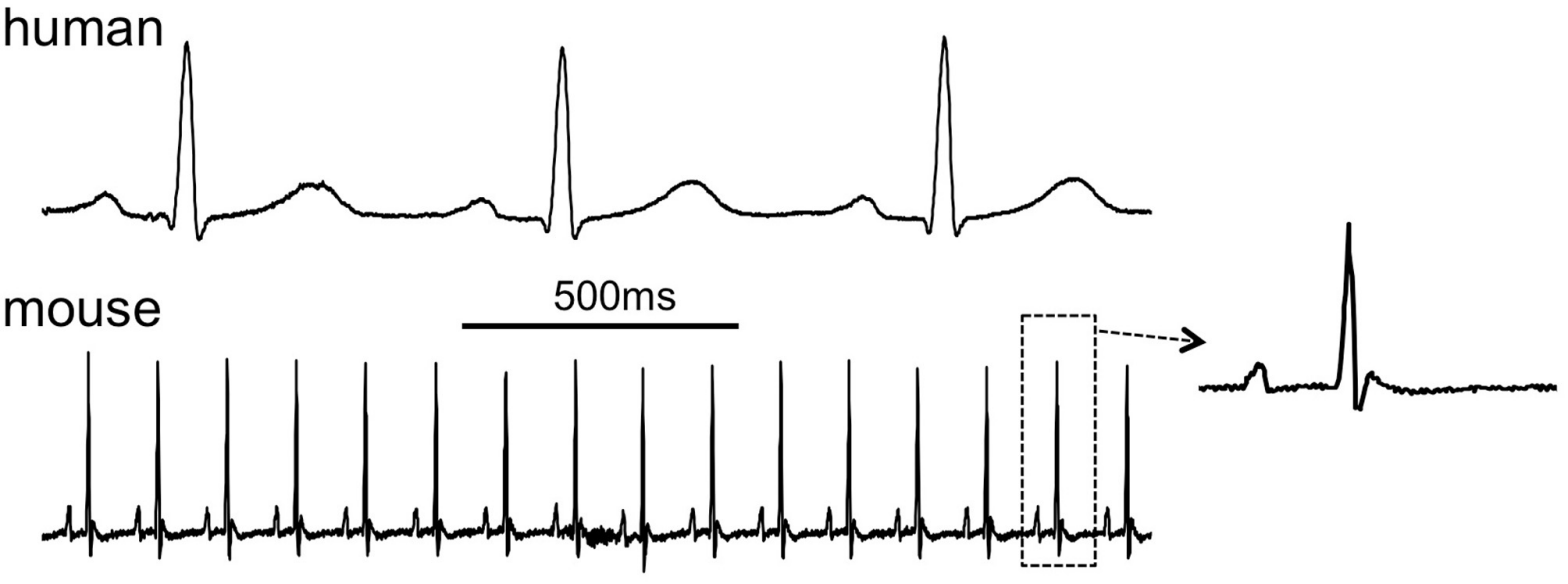
**Comparison of the human and murine ECG, 2-s traces showing lead I.** Inset shows a single complex from the mouse ECG (human ECG is patient 121 in the PTB database at www.physionet.org, mouse ECG courtesy of Dr. Ricardo Carnicer, Oxford University).

Over the entire range of BM form mice to whales, cardiac mass amounts 0.6% of body mass (Prothero, 1979) and cardiac gross anatomy is remarkably similar, with comparable pacemaking and conduction structures (Wessels and Sedmera, 2003). Using the same biochemical processes to ensure optimal electromechanical function, hearts of very different sizes have to maintain sufficient cardiac function under a variety of conditions. Resting heart rates in conscious mice (measured by telemetry) range from 550 to 725 beats per minute (bpm) (Kass et al., 1998; Gehrmann et al., 2000; Janssen and Smits, 2002; Fabritz et al., 2010), corresponding to a sinus cycle length (SCL) of 80–110 ms. Because cardiac conduction velocities are not strongly dependent on BM (Table 1 and Figure 1), the timing intervals of the ECG scale with the propagation distance, i.e., cardiac size. The ECG time intervals RR, PR, QRS, and QT scale with BM^1/4^ (Noujaim et al., 2004, 2007) and consequently heart rate scales with body mass with an exponent of -1/4 (West et al., 1997; Janssen and Smits, 2002; Noujaim et al., 2004). The PR interval represents electrical conduction from the atrium through the AV node and His-Purkinje system to the ventricles and thus can be seen as a fractal network with three separate components, the PA, AH, and HV subintervals, that also scale with body mass with an exponent of 1/4 (Noujaim et al., 2004). Hence, percentage of each subinterval to the PR interval is constant and independent of body and heart size (Noujaim et al., 2004).

Mice can increase their HR by around 30–40% (Vornanen, 1992; Kass et al., 1998; Fabritz et al., 2010), whereas humans HR can increase by 300%. Thus, an increase in frequency can contribute far less to increase cardiac output (CO) in mice than in humans. The ratio of maximal to resting heart rate scales to BM with the following equation HR_max_/HR_rest_ = 1.87 × BM^−0.1^ (Kass et al., 1998). Actin-myosin crossbridge kinetics have an estimated maximal rate of around 1000 bpm, corresponding to a cycle length (CL) of 60 ms (Kass et al., 1998). Maximal heart rates observed in mice are in a range of 725–815 bpm (Vornanen, 1992; Kass et al., 1998; Fabritz et al., 2010). Thus, an increase in murine HR is limited as the maximal HR is close to the maximal actin-myosin crossbridge frequency. In addition, studies in mice have found an AV Wenckebach period of 66–103 ms (Table 1). With an increase in HR, this Wenckebach period is reached relatively quickly, also limiting the increase in CO. For efficient cardiac pump function and CO, optimal timing of AV conduction is required. Late diastolic left ventricular filing time scales with heart length and with an 1/3 exponent to cardiac or body mass, whereas AV delay scales with an ¼ exponent of heart or body mass (Noujaim et al., 2004; Meijler et al., 2005). In mice, an increase in heart rate decreases diastolic left ventricular filing time and thus gradually reduces ventricular end-diastolic volume. Finally, mice at physiological HR have a small force-frequency reserve compared to humans (Georgakopoulos and Kass, 2001). Consequently, ventricular filing is limited at high heart rates in mice, further impeding CO enhancement and cardiac contractility. Taken together, these factors explain why mice can raise their CO only over a small range, whereas humans can increase their CO by a factor 5–6. This difference should be taken into account when HR regulation (e.g., during exercise) is relevant.

## Cardiac activation in mice

### Sinoatrial node

The lateral and medial limb of the crista terminalis at the orifice of the superior caval vein frame the murine SA node region (Verheijck et al., 2001; Mommersteeg et al., 2007). During development, the SA is node is distinguished from the adjacent atrial myocardium by the absence of the transcription factor Nkx2.5 and the presence of the transcription factors Tbx3 and Tbx18, both in humans and mice (Christoffels et al., 2006; Mommersteeg et al., 2007; Sizarov et al., 2011). These differences in transcription factor profiles initiate diverging gene expression programs, leading to differences in e.g., connexin and ion channel expression (Hoogaars et al., 2007). Histologically, the SA node consists of a large head and a smaller tail along the crista terminalis (Wiese et al., 2009). Characteristic sinus node APs (a marked diastolic depolarization and low upstroke velocity) are found in a region 300 μm in parallel and 150 μm perpendicular to the crista terminalis (Opthof, 2001; Verheijck et al., 2001). A morphologically “nodal” cell type is present in a somewhat larger region 500 μm parallel to the crista (Opthof, 2001; Verheijck et al., 2001). Surprisingly, Glukhov et al. have shown that in the mouse, the location of the dominant pacemaker can shift during (para)sympathomimetic treatment within a still larger region of approximately 5 × 2 mm between the superior and inferior caval veins (Glukhov et al., 2010).

The sinoatrial conduction time, defined as the conduction time from the primary, dominant sinus node cells to atrial myocardium is 4–5 ms and the transition from a nodal to an atrial AP morphology takes place over a very short distance of about 100 μm (Opthof, 2001; Verheijck et al., 2001).

SA nodal APs in mice have an amplitude, maximum diastolic potential and upstroke velocity comparable to those in humans, but a much shorter APD, probably due to differences in delayed rectifier currents (Opthof, 2001). The SCN5A encoded cardiac Nav1.5 sodium channel affects SA node pacemaker function, as heterozygous deletion of the SCN5A gene in mice caused sinus bradycardia and exit block (Lei et al., 2005). Similarly, sinus bradycardia and sinus pauses are prevalent in LQT3 patients with certain SCN5A mutations (Veldkamp et al., 2003). However, SCN5A-related alterations in sinus node function may be caused by the involvement of Nav1.5 in extracellular matrix remodeling, rather than a primary electrical effect (Hao et al., 2011). The α_1D_ form of the L-type Ca^2+^ channel is expressed in the SA node and contributes to diastolic depolarization and pacemaker activity, and deletion of this gene leads to sinus bradycardia (Zhang et al., 2002; Mangoni et al., 2003; Qu et al., 2005).

The murine SA node expresses hyperpolarization-activated, nonselective cation channels from the HCN family. HCN4 is the dominant isoform, although smaller amounts of HCN1 and HCN2 are also expressed (Liu et al., 2007; Ludwig et al., 2008; Herrmann et al., 2011). Specific mutations in the HCN4 gene can cause bradycardia in humans (Milanesi et al., 2006). HCN4 knock-out mice have produced diverging results (Herrmann et al., 2012). Mice with conditional ubiquitous HCN4 deletion in the adult stage were not bradycardic, even at less than 10% of normal HCN4 levels, but did show sinus pauses that were activity dependent and more prevalent at low heart rates (Herrmann et al., 2007; Hoesl et al., 2008). I_f_ in isolated SA nodal cells from these mice was decreased by 75% and these cells often did not show pacemaker activity under baseline conditions, but did become spontaneously active in response to adrenergic stimulation (Herrmann et al., 2007; Ludwig et al., 2008). However, another mouse model with heart-specific conditional deletion of HCN4 did show profound sinus bradycardia (Baruscotti et al., 2011). HCN2 deficient mice have a sinus dysarrhythmia leading to a marked increase in RR variability, although the response to adrenergic and cholinergic stimulation was preserved (Ludwig et al., 2003).

In mice, Cx45 is expressed in SA node center (Opthof, 2001; Verheijck et al., 2001). On the epicardial side, the murine SA node is entered by strands of cells expressing Cx40 and Cx43 (Verheijck et al., 2001). Also in humans, atrial myocardial cells with Cx43 expression protrude into the Cx43 negative SA nodal area (Oosthoek et al., 1993). The murine SA node architecture containing different types of gap junctions and connective tissue around the nodal area are hypothesized to shield the SA node against hyperpolarizing influence of surrounding atrial myocardium and thus to allow pacemaker activity and conduction from the SA node to atrial myocardium (Verheijck et al., 2001).

### Atrial activation

Various studies have shown atrial conduction patterns in mice. Atrial activation starts near the expected location of the sinoatrial node close to the superior vena cava and from there spreads rapidly over the right and left atria (Leaf et al., 2008; Mathur et al., 2009; Blana et al., 2010; Kirchhof et al., 2011; Swaminathan et al., 2011). It is not clear whether mice have a true Bachmann's bundle, which in larger species is a thick, highly anisotropic structure consisting of many parallel fibers, serving as the largest and fastest conduction pathway between the atria. The total atrial conduction time amounts to approximately 15 ms (Blana et al., 2010; Swaminathan et al., 2011). The atria express both Cx40 and Cx43. The specific role of Cx40 is unclear, with observations in Cx40^−/−^ mice ranging from a reduced CV (Verheule et al., 1999) to a reduced conduction heterogeneity without a change in CV (Leaf et al., 2008). The atrial ERP shows little rate-dependence and in some cases it may be longer than the AV nodal ERP (Etzion et al., 2008).

### Atrioventricular node

The murine AV node has similar properties to the human AV node (VanderBrink et al., 1999). In some humans, the AV node shows “dual AV physiology” with a distinct slow and fast pathway, giving rise to a discontinuous AV conduction curve during premature atrial stimulation. The prevalence of dual AV nodal physiology increases during postnatal development and around 40% of adult humans display dual AV nodal physiology (Denes et al., 1973; Thapar and Gillette, 1979; Cohen et al., 1997). A maturation study in mice demonstrated that dual AV nodal physiology and AV nodal echo beats also become more frequent with increasing age, but without inducibility of AV nodal reentry tachycardia (Maguire et al., 2000). In contrast, another study of the murine AV node did not find dual AV nodal physiology (VanderBrink et al., 1999). As in larger species, the AV node shows “facilitation” during an acceleration in rate, and this property is lost in Cx40 deficient mice (Zhu et al., 2005).

The areas bordering the AV node show a complex profile of Cx expression, which has been studied in mice and humans (Aanhaanen et al., 2010; Greener et al., 2011). Myocytes in the compact node, the area responsible for the major part of the AV delay, express Cx40 and Cx45 in both mice and humans. In addition, Cx30.2 is expressed in mice, but its orthologue Cx31.9 is not present in the human AV node. In Cx40^+/−^ mice and Cx45^+/−^ mice, AV conduction is not affected (Hagendorff et al., 1999; Verheule et al., 1999; Kruger et al., 2006). The AV delay increases in Cx40^−/−^ mice (Hagendorff et al., 1999; Verheule et al., 1999) and increases further in Cx40^−/−^Cx45^+/−^ mice (Kruger et al., 2006), indicating that both connexins contribute to AV conduction. Intriguingly, the AV delay is shorter in Cx30.2^−/−^ mice and normal in Cx30.2^−/−^Cx40^−/−^ mice (Kreuzberg et al., 2006; Schrickel et al., 2009). Thus, Cx30.2 may represent an arrangement to decelerate AV nodal conduction that is specific to mice.

In humans, the homeobox transcription factor Nkx2-5 is expressed in the AV node and heterozygous mutations caused congenital atrioventricular conduction abnormalities, e.g., AV block (Schott et al., 1998). A murine model with a heterozygous Nkx2-5 mutation has a hypoplastic AV node (Jay et al., 2004). Furthermore, overexpression of a human mutation of Nkx2-5 in transgenic mice led to heart failure and progressive AV conduction defects, possibly due to reduced Cx40 and Cx43 expression (Kasahara et al., 2001). Similarly, haploinsuffiency in mice of the transcription factor Tbx5, which has a synergystic effect with Nkx2-5 on gene transcription, causes abnormalities similar to those in the human Holt-Oram syndrome, including decreased Cx40 expression and AV block (Bruneau et al., 2001; Moskowitz et al., 2007).

### Ventricular activation and repolarization

The ventricular conduction system lineage is established during development by expression of the transcription factors Nkx2-5 and Tbx5, (Jay et al., 2004; Moskowitz et al., 2007) activating a gene program that includes Cx40, the major gap junction protein in the His bundle and proximal conduction system. Deletion of Cx40 leads to conduction slowing in the ventricular conduction system (Simon et al., 1998; Bevilacqua et al., 2000; VanderBrink et al., 2000). The murine left bundle branch is thicker than the right bundle branch, as evidenced by Cx40 expression patterns (van Rijen et al., 2001). This is supported by studies that suggested that the left bundle brunch contains more parallel fibers and thus probably has a higher conduction reserve than the right bundle branch, possibly explaining why the right bundle branch is more prone to conduction block (Alcolea et al., 2004). Similarly, clinical studies tend to show a higher prevalence of conduction block in the right bundle branch than in the left bundle branch (Tuzcu et al., 1990; Newby et al., 1996; Golshayan et al., 1998).

The activation pattern of ventricular myocardium differs between mice and humans. In mice, on study has reported that ventricular epicardial activation starts with clearly defined breakthroughs in the right ventricle and subsequently in the left ventricle (Tamaddon et al., 2000). Another study has shown a first breakthrough at the left ventricular apex, followed shortly by a right ventricular breakthrough (Nygren et al., 2000). By contrast, studies in humans found first depolarization at the left ventricular side of the interventricular septum, conducted by septal left bundle branch fibers and corresponding to the Q wave in the ECG (Durrer et al., 1970). Septal right bundle branch fibers cause subsequent right ventricular activation (Durrer et al., 1970). Within the interventricular septum, mice show an activation pattern from base to apex, probably due to an electrical connection from the common bundle into the ventricular septum (van Rijen et al., 2001). By contrast, humans show intraventricular septal activation from left to right and from apex to base (Durrer et al., 1970). Conduction velocity is slower in the midseptal than in the proximal region of the murine bundle branches, due to regional differences in bundle geometry and connexin expression (van Veen et al., 2005). During murine intrauterine development, the epicardial ventricular activation pattern switches from basico-apical to apico-basal, in correspondence with maturation of the conduction system (Rentschler et al., 2001; Gourdie et al., 2003).

Within the mouse ventricles, repolarization is earlier in the epicardium than in the endocardium due to a shorter epicardial action potential (Knollmann et al., 2001), and earlier at the apex than at the base (Killeen et al., 2007b; London et al., 2007). Transmural and apico-basal gradients in I_to_ are thought to be the main determinant of heterogeneity of the murine ventricular APD (Rossow et al., 2006; Wang et al., 2006; Killeen et al., 2007b; London et al., 2007).

Cx43 is the main gap junction protein in the ventricular working myocardium, but its expression can be decreased by ~80% before a marked decrease in CV and increase in vulnerability to arrhythmias becomes apparent (Danik et al., 2004; van Rijen et al., 2004), as would be expected based on mathematical models (Shaw and Rudy, 1997). However, more modest reductions in gap junctional coupling may have significant effects when they occur in conjunction with other factors such as reduced excitability or fibrosis (van Veen et al., 2005; Stein et al., 2009). Similar conjunctions of disease processes are common in human pathology, but poorly represented by monogenic mouse models.

### Arrhythmias linked to abnormal cardiac development

Some studies have linked human arrhythmias to gene programs active during prenatal cardiac development. The developing mouse heart contains muscular atrioventricular connections over the annulus fibrosus (Rentschler et al., 2001). Persistence of these connecting fibers bypass the AV node and cause ventricular pre-excitation syndromes similar to Wolff-Parkinson-White syndrome (Rentschler et al., 2001) Patients suffering from Wolff-Parkinson-White syndrome showed mutations in the PRKAG2 gene, which encodes the gamma-2 subunit of the AMP-activated protein kinase (Blair et al., 2001; Gollob et al., 2001). In mice, overexpression of one of the human mutations of the PRKAG2 gene caused ventricular preexcitation and an altered annulus fibrosis structure, disrupted by glycogen-filled myocytes that may function as accessory pathways (Arad et al., 2003). Similarly, inactivation of Tbx2, a transcription factor involved in the development of the atrioventricular canal, leads to malformation of the annulus fibrosis and ventricular preexcitation (Aanhaanen et al., 2011). Mutations in Tbx3 are linked to ulnar-mammary syndrome in humans. In mice, a reduction in Tbx3 activity leads to SA node dysfunction, AV block and ventricular preexcitation (Frank et al., 2012).

Gittenberger-de Groot and co-workers have presented extensive evidence for the relationship between nodal pacemaker tissue and the myocardial sleeves in the developing embryo (Douglas et al., 2011) For example, podoplanin is a myocardial marker expressed in cells of the developing pacemaking system, which later differentiate e.g., to the sinoatrial node (SA node) and atrioventricular node (AV node) (Gittenberger-de Groot et al., 2007). Podoplanin expression is also present in surrounding myocardium of the common pulmonary vein (PV) (Gittenberger-de Groot et al., 2007), an area that can later become important in the genesis of atrial fibrillation in humans (Haissaguerre et al., 1998). These embryological relationships have been proposed account for ectopic activity in the myocardial sleeves of the pulmonary veins (Jongbloed et al., 2004). However, the sinus node originates from a different type of cardiac precursor cells (i.e., Tbx18-positive and Nkx2.5-negative) than the PV myocardium (Christoffels et al., 2006). In addition, a nodal phenotype would be more likely to give rise to relatively slow, gradually appearing and disappearing pacemaking activity, as has been described in guinea pig pulmonary veins (Cheung, 1981). By contrast, human PVs show paroxysms of arrhythmic activity that initiate and terminate suddenly, with very rapid rates that are more compatible with triggered activity or microreentry than with a pacemaking activity in a latent nodal region (Haissaguerre et al., 1998).

## Cardiac cellular electrophysiology

In general, the duration of cardiac action potentials increases with body size and is approximately 50 ms in mouse ventricles (Danik et al., 2002), compared to 250 ms in humans (Edvardsson et al., 1984). The morphology of the action potential reflects the contribution of numerous depolarizing and repolarizing currents. Even when the same type of ion channel is expressed in human and mice, its contribution to the morphology of the action potential may differ substantially, given the large difference in APD. Both in mice and humans, the refractory period ends before complete repolarization of the AP (Knollmann et al., 2001; Fabritz et al., 2003; Sabir et al., 2007a,b). Based on recordings of monophasic action potentials, the ERP in mouse ventricles corresponds to the APD80 (Knollmann et al., 2001).

Murine and human ventricular APs are both characterized by a fast depolarizing phase (phase 0), the AP upstroke, which is generated by the large, rapidly activating sodium current I_Na_ (Nerbonne, 2004). The subsequent repolarization phase is a delicate balance of several depolarizing currents (I_CaL_, I_NCX_) and repolarizing potassium currents. In the human ventricle, a fast repolarizing phase (phase 1) is followed by the action potential plateau (phase 2) (Nerbonne, 2004). In the mouse heart, the L-type Ca^2+^ current (I_CaL_) contributes less to the ventricular AP than in humans (Sabir et al., 2008) and therefore the murine AP shows a gradual repolarization rather than a distinct plateau phase. In the mouse ventricles, α_1C_ channels are primarily responsible for I_CaL_. By contrast, the I_CaL_ of supraventricular myocardium consists of both α_1C_ and α_1D_ Ca^2+^ channels (Zhang et al., 2005; Mancarella et al., 2008; Zhang et al., 2011). The α_1D_ Ca^2+^ channel makes a significant contribution to Ca^2+^ influx and Ca^2+^ induced Ca^2+^ release from the SR in atrial myocytes (Mancarella et al., 2008).

In humans, the plateau phase ends when the balance shifts from I_NCX_ and the slowly inactivating I_CaL_ to slowly activating potassium currents, giving rise to the final repolarization (phase 3) until the resting potential (phase 4) is restored. In the human ventricle, the rapid and slow delayed outward rectifier K^+^ currents (I_Kr_ and I_Ks_) are predominantly responsible for phase 3 repolarization (Li et al., 1996). In contrast, the much faster repolarization in mice ventricles is mediated by transient outward K^+^ currents with a fast and slow recovery from inactivation (I_to,f_ and I_to,s_), a slowly inactivating K^+^ current (I_K,slow1_ and I_K,slow2_) and a non-inactivating, steady state current (I_ss_) (Guo et al., 1999; Xu et al., 1999; Zhou et al., 2003; Brouillette et al., 2004; Sabir et al., 2008). In humans, the I_to,f_ current is mainly involved in phase 1 repolarization, with more prominent expression in the epicardium (Nerbonne, 2004). In mice, I_to,f_ is expressed throughout the left and right ventricles, whereas I_to,s_ is confined to the septal myocardium (Xu et al., 1999). The equivalents of murine I_ss_ and I_Kslow_ have not been detected in human ventricles (Sabir et al., 2008). Studies in mice did observe the rapid and slow delayed rectifier K^+^ currents (I_Kr_ and I_Ks_), but their contribution to repolarization under physiological conditions is probably negligible or minor (Babij et al., 1998; Drici et al., 1998; Balasubramaniam et al., 2003; Nerbonne, 2004; Salama et al., 2009). In the ventricles of mice and humans, the inwardly rectifying K^+^ channel (I_K1_) plays a similar role in stabilizing the resting membrane potential and determining terminal repolarization (Babenko et al., 1998; Flagg and Nichols, 2001; Lopatin and Nichols, 2001; Nerbonne et al., 2001; Nerbonne, 2004). During postnatal development, the APD shortens progressively as a result of the upregulation of a number of potassium currents, both in the atria (Trepanier-Boulay et al., 2004) and in the ventricles (Grandy et al., 2007). In the adult mouse heart, atrial and ventricular action potentials are very similar in morphology [for a detailed review of all underlying currents see Nerbonne (2004)].

Genetically engineered mice have revealed surprising contributors to the cardiac action potential. For example, deletion of the calcium-activated potassium channel SK2 prolongs the APD in atrial myocytes and leads to AF (Li et al., 2009), although SK channels do not seem to contribute to the normal atrial action potential in larger species (Nagy et al., 2009). SK channel function may be affected by the tendency of SK subunits to form heteromers (Tuteja et al., 2010), and its functional contribution may increase under pathological conditions, also in the ventricular myocardium (Chua et al., 2011). In the mouse ventricles, deletion of the “pacemaker current” HCN3 leads to an increase in T wave amplitude due to an acceleration of the terminal repolarization in epicardial myocytes (Fenske et al., 2011).

The main Ca^2+^ extrusion mechanism in cardiomyocytes is the Na^+^/Ca^2+^ exchanger (NCX), which generates a net depolarizing current due to its stoichiometry (Bers, 2002; Pott et al., 2004). In most respects, mice overexpressing NCX and cardiac-specific NCX knockouts show opposite cardiac phenotypes, although cardiac hypertrophy was observed in both (Henderson et al., 2004; Pott et al., 2004; Goldhaber et al., 2005; Reuter et al., 2005; Pott et al., 2012). Due to the calcium-dependence of I_CaL_ inactivation, NCX overexpressing mice and cardiac-specific NCX knockouts had accelerated and decelerated inactivation kinetics of I_CaL_, respectively, leading to APD prolongation and shortening, respectively (Pott et al., 2004; Goldhaber et al., 2005; Reuter et al., 2005; Pott et al., 2007). NCX overexpression increased and cardiac-specific NCX deletion decreased vulnerability to ischemia/reperfusion injury, supporting the role of calcium influx through the reverse-mode NCX under these circumstances (Pott et al., 2004; Imahashi et al., 2005). In hearts of larger mammals, like dog, rabbit, and human, reuptake into the sarcoplasmic reticulum and elimination by the NCX account for approx. 70% and 30% of Ca^2+^ removal, respectively, while in mice and rats up to 90% of Ca^2+^ is taken back up into the sarcoplasmic reticulum (Bassani et al., 1994; Nishimaru et al., 2001). Under steady state conditions (i.e., a stable diastolic SR calcium load), the amount of calcium extruded by the sodium-calcium exchanger (NCX) equals the amount of calcium entering through L-type calcium channels. Increased NCX expression is thought to contribute to arrhythmogenesis in failing human and rabbit hearts (Studer et al., 1994; Lindner et al., 1998; Pieske et al., 1999; Pogwizd et al., 1999, 2001; Schillinger et al., 2000), and EADs, DADs and episodes of ventricular tachycardia were inducible in NCX-overexpressing mice (Pott et al., 2012). Increased NCX expression causes reduced twitch calcium transients and SR calcium load in rabbit ventricles (Ranu et al., 2002). In contrast, increased NCX expression in mice does not affect twitch Ca^2+^ transients and SR calcium load (Adachi-Akahane et al., 1997; Terracciano et al., 1998; Yao et al., 1998). This difference between mice and larger mammals may be caused by a higher cytoplasmic sodium concentration ([Na]_i_] in murine myocytes (Shattock and Bers, 1989; Yao et al., 1998; Despa et al., 2002), which would impair extrusion via NCX (Bers, 2002). A negative force-frequency relationship has been reported for murine ventricles, often with high Ca^2+^ concentration of the SR, even under low stimulation frequencies (Bers, 2002). Again, the high [Na]_i_ may impair Ca^2+^ extrusion via NCX and explain why an increasing in heart rate hardly affects SR calcium load in mice (Shattock and Bers, 1989; Yao et al., 1998). In addition, refractoriness of excitation contraction coupling and smaller fractional SR Ca^2+^ is observed during high heart rates (Bers et al., 1993). Both mechanisms probably contribute to negative force-frequency relationship in mouse ventricular myocytes (Bers, 2002).

## Changes in cardiac electrophysiology in mouse models of heart disease

### Myocardial infarction

Mouse models of myocardial ischemia and infarction are commonly generated by ligation of the left anterior descending coronary artery (LAD). Temporary ligation of the LAD with subsequent reperfusion has been used as a model of ischemia-reperfusion injury, allowing determination of the infarct size in relation to the area at risk (Michael et al., 1995). In Langendorff-perfused mouse hearts, regional ischemia and reperfusion increased the incidence of induced and spontaneous ventricular tachycardia (VT) (Koyama et al., 2003; Inagaki et al., 2005; Anzawa et al., 2006; Maass et al., 2009; He et al., 2012). Depending on the exact inschemia-reperfusion protocol, ventricular fibrillation (VF) may also be observed (Koyama et al., 2003; Inagaki et al., 2005; He et al., 2012), especially when catecholamines are added to the perfusate (Stables and Curtis, 2009). In open-chest experiments, episodes of ventricular tachycardia (VT) were observed both during regional ischemia and reperfusion (Sakamoto et al., 1999; Anyukhovsky et al., 2011). Mice with a permanent ligation of the LAD show infarct-related changes in T-wave morphology, reduced cardiac function, and increased inducibility of atrial and ventricular arrhythmias (Gehrmann et al., 2001). In conscious mice with a myocardial infarction (MI), telemetric monitoring revealed frequent premature ventricular beats, but spontaneous VT episodes were rare (Betsuyaku et al., 2004). Heart rate variability (HRV) a marker of autonomic nervous system activity and a predictor of arrhythmias in human MI patients, was not affected by MI in mice (Gehrmann et al., 2001) Decreased HRV is thought to result from an enhanced sympathetic and decreased parasympathetic activity, but in contrast to humans, mice already show a predominant sympathetic tone, since parasympathetic blockade does not increase in HR (Mansier et al., 1996; Wickman et al., 1998) but sympathetic blockade caused a pronounced decrease in HR (Janssen et al., 2000; Just et al., 2000). For a detailed review on autonomic regulation of cardiac function in mice, see (Janssen and Smits, 2002).

### Pressure overload

Transverse aortic constriction (TAC) by banding the proximal aorta has become a standard method to generate chronic pressure overload in mice, leading to LV hypertrophy with decreased fractional shortening within 4–5 weeks (Hill et al., 2000). TAC leads to an increase in QRS duration and a decrease in RV longitudinal conduction velocity (Boulaksil et al., 2010). Furthermore, these mice showed increased interstitial fibrosis and Cx43 heterogeneity (Boulaksil et al., 2010), and an increased inducibility of polymorphic ventricular tachyarrhythmias (Boulaksil et al., 2010; Jansen et al., 2012; Vinet et al., 2012). The inducibility and stability of atrial fibrillation also increase as a result of TAC (Liao et al., 2010). In addition to the working myocardium, pressure overload may also affect Cx and HCN expression in the ventricular conduction system and thereby alter the ventricular activation pattern (Harris et al., 2012).

## Methods for studying murine cardiac electrophysiology

Different mouse strains show considerable differences in baseline electrocardiographic parameters (Wehrens et al., 2000; Waldeyer et al., 2009) and in processes such as for example wound healing after myocardial infarction (van den Borne et al., 2009), underscoring the importance of a homogeneous genetic background in assessing the role of a genetically modified gene. Studying murine cardiac physiology has required the miniaturization of instruments and techniques originally developed for human studies. This section will briefly outline a number of techniques that are currently available.

### Surface electrocardiograms

As shown in Figure 1, murine and human ECGs show some salient differences that complicate comparison of ECGs from both species (Sabir et al., 2008). In both species, the ECGs show P waves, reflecting spread of atrial depolarization (Sabir et al., 2008). Subsequently, both ECGs show an isoelectric PQ interval that reflects impulse conduction from the atria through the AV node and the His-Purkinje system to the ventricles (Schrickel et al., 2002; Sabir et al., 2008). In mice and humans, the ensuing QRS complex reflects ventricular depolarization (Schrickel et al., 2002; Sabir et al., 2008). The normal murine ECG shows a distinct J wave at the end of the QRS complex (Liu et al., 2004). By contrast, the human ECG infrequently displays a slight J wave, but it can become more prominent during hypothermia (Osborn, 1953). Because of the triangular shape of the murine ventricular action potential, with a gradual time course of repolarization, mouse ventricles do not have a distinct “moment of repolarization.” As a result, the T wave in the murine ECG is often poorly visible (Liu et al., 2004; Sabir et al., 2008). In some murine studies, the QT interval was defined as the period between the start of the Q wave to the point of return to the isoelectrical line (Verheule et al., 1999; Schrickel et al., 2002). However, correlation of monophasic action potential recordings with the surface ECG indicates that the terminal repolarization of the ventricles may extend into the subsequent P-wave (Danik et al., 2002; Liu et al., 2004). The absence of a clear T wave impedes investigation of murine ventricular repolarization in the surface ECG (Danik et al., 2002). Within the physiological range, the relative degree of rate dependence of the ventricular APD is comparable between mice and humans (Knollmann et al., 2007). The murine QT interval can be corrected for heart rate using Bazett's formula, which is used in human ECG analysis, or (preferably) by a related formula specifically modified for mice (Mitchell et al., 1998).

Small implantable ECG transmitters permit investigation of spontaneous arrhythmias in conscious mice during normal physical activity and during short-term and long-term drug application (Gehrmann and Berul, 2000; Gehrmann et al., 2000; Fabritz et al., 2010). This technique also allows investigation of acute and chronic exercise stress on arrhythmogenesis by using standardized protocols such as e.g., swimming exercise (Berul, 2003; Fabritz et al., 2010). In determining the degree of exercise, it should be take into account that mice will voluntary run a respectable distance of 6 km per day (de Waard et al., 2007).

### Programmed electrical stimulation in intact mice

In intact mice under anaesthesia, transesophageal catheters can be used for programmed electrical stimulation of the atria for arrhythmia induction and determination of sinus node recovery time, AV nodal ERP and Wenckebach period (Schrickel et al., 2002; Berul, 2003; Verheule et al., 2004). Due to the lack of direct contact with the atrium and the distance between stimulation poles, stimulus artefacts during transesophageal stimulation are large, obscuring the atrial complex. Therefore, the atrial ERP can not be determined reliably using this technique (Etzion et al., 2008). Because transesophageal stimulation allows survival of the animal, it can be used for longitudinal studies of cardiac electrophysiology over a longer time period. The ventricles cannot be paced using transesophageal stimulation, but Gutstein et al. have presented a method for subdiaphragmatic ventricular stimulation that allows repeated studies in individual mice (Gutstein et al., 2003).

Pacing (and recording) electrodes can be attached either epicardially in open chest experiments (Berul et al., 1996; Verheule et al., 1999) or endocardially using a transvenous access route (VanderBrink et al., 1999; Saba et al., 2000). The latter also permits the recording of his bundle potentials (VanderBrink et al., 1999; Saba et al., 2000). Programmed electrical stimulation using epi- or endocardial electrodes allows assessment of SA node and AV node function, refractory periods, Wenckebach periodicities and vulnerability to atrial and ventricular arrhythmias (Gehrmann et al., 2000; Saba et al., 2000; Berul, 2003). Given the small dimensions of the mouse heart, close apposition of the poles of the stimulation electrode is necessary to reduce stimulus artefacts and allow recording of local electrograms (Verheule et al., 1999; Etzion et al., 2008).

### Mapping in perfused mouse hearts

Isolated Langendorff perfused hearts allow detailed studies of cardiac electrophysiology without neurohumoral and autonomic influences, under artificial conditions that can easily be manipulated. Perfused hearts allow pacing and recording of electrograms and monophasic action potentials (Fabritz et al., 2003; Blana et al., 2010). In addition, the Langendorff setup offers the opportunity to perform high-density epicardial mapping using multi-electrode arrays to record activation patterns (van Rijen et al., 2001; Verheule et al., 2004). Sampling frequency has to be sufficiently high in mapping experiments. For example, with a relatively simple 5 × 5 electrode array covering 2 × 2 mm (about the size of an atrial free wall), the inter-electrode distance would be 0.4 mm. Assuming a conduction velocity of 0.5 m/s, (Verheule et al., 1999, 2004) the time difference between electrodes in the propagation direction would be 0.8 ms. Accurate determination of local conduction velocities would then require a sampling frequency of at least 2.5 kHz (1/0.8 × 2).

With currently available technology, the highest spatio-temporal resolution can be attained using optical mapping (up to 100 × 100 pixels at a sampling rate of up to 10 kHz). Using this technique, detailed activation mapping has been reported in various mouse models (de Diego et al., 2008; Blana et al., 2010; Lang et al., 2011). In addition, the acquired optical signals are linearly dependent on the transmembrane potential and therefore represent an “ensemble action potential,” providing information on the time course of repolarization and diastolic intervals. Furthermore, activation patterns during arrhythmias, e.g., rapid reentry, can be studied in detail (Vaidya et al., 1999; Baudenbacher et al., 2008). However, to suppress motion artefacts, most studies have used excitation-contraction uncouplers such as butadione monoxime, cytochalasin D or blebbistatin, all of which can affect cardiac electrophysiology in mice. 2,3-Butadione monoxime (BDM) markedly shortens the APD (Biermann et al., 1998). In mice, Cytochalasin D prolongs APD and reduces CV (Baker et al., 2004). Blebbistatin has little effect on the AP morphology, (Fedorov et al., 2007) but can suppress arrhythmias by inhibiting myofilament calcium sensitivity (Baudenbacher et al., 2008).

## The issue of cardiac size in tachy-arrythmias

In 1914, Garrey postulated that a certain size of myocardial tissue, a “critical mass,” is required to sustain reentrant arrhythmias (Garrey, 1924). This led to the belief that the mouse heart would be “too small to fibrillate.” This belief has been challenged by murine studies showing atrial (Schrickel et al., 2002; Verheule et al., 2004) and ventricular fibrillation (Vaidya et al., 1999). Specific mouse models of atrial (Nattel et al., 2005; Schotten et al., 2011) and ventricular (Sabir et al., 2008) arrhythmias have been expertly reviewed elsewhere. Similarly, the general mechanisms of atrial (Nattel et al., 2005; Schotten et al., 2011) and ventricular (Antzelevitch, 2001) arrhythmias have been reviewed extensively. Instead, this last section will discuss the general issue of cardiac size in the context of arrhythmias. With respect to the propagation pattern, arrhythmias can be either “hierarchical,” with one or more localized sources driving the arrhythmia or “anarchical” where no particular site is driving the arrhythmia. Whereas the former is amenable to targeted ablation strategies, the latter is not. With respect to the underlying mechanism, an arrhythmia will be based on cellular proarrhythmic events giving rise to premature, ectopic action potentials or on reentrant conduction (Antzelevitch, 2001; Sabir et al., 2008). Regardless of the system of classifying arrhythmias, in all cases the size of the substrate is likely to affect the initiation, stability and pattern of the arrhythmia, as discussed below.

### Reentry

**Figure 2:**
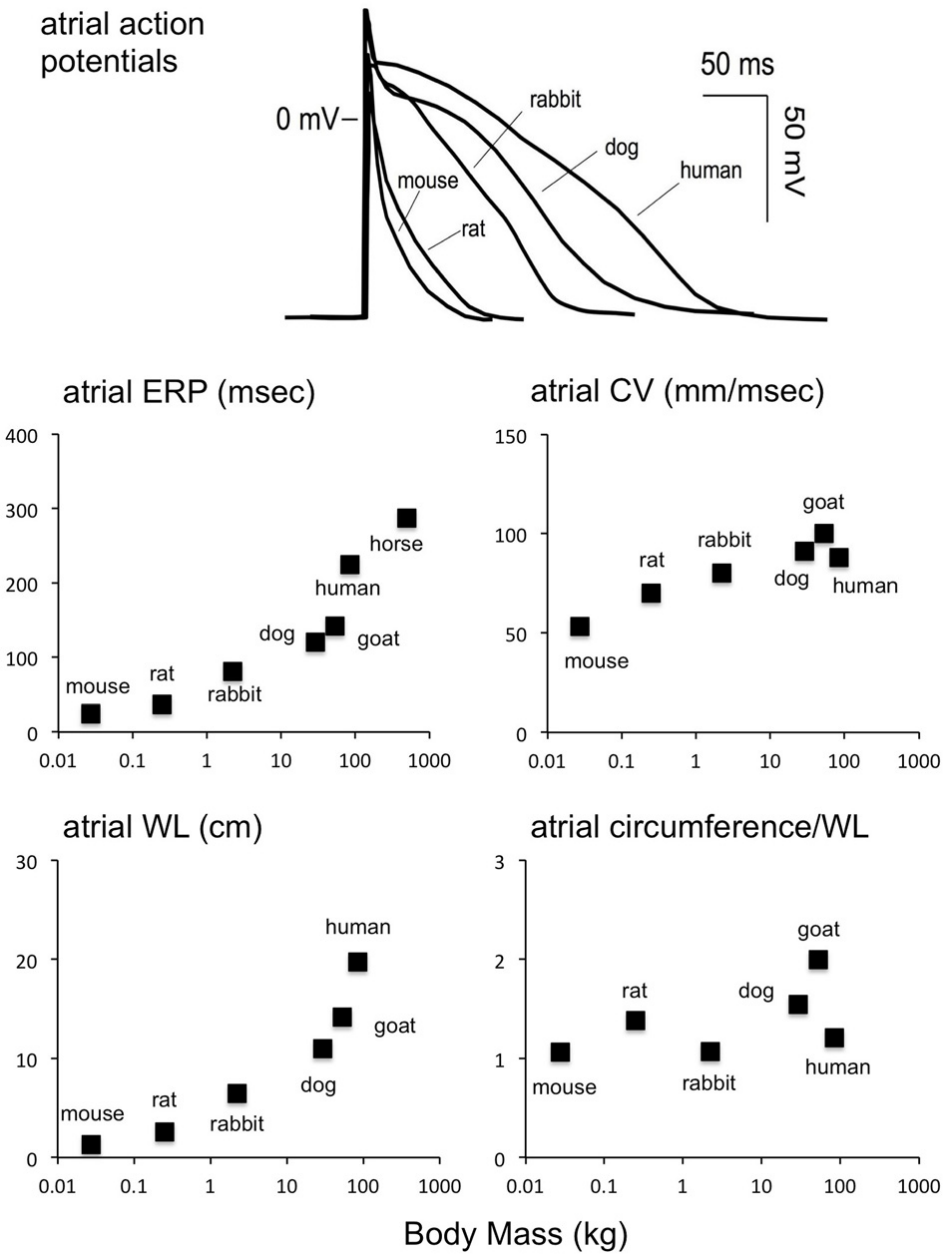
**Scaling of atrial electrophysiology to body mass.** Upper panel: atrial action potentials in various species (Schotten et al., 2011). Middle row, left panel: atrial ERP as a function of body mass (mouse Verheule et al., 1999, rat Chang et al., 2002, rabbit Fedorov et al., 2007, dog Fareh et al., 1998, goat Blaauw et al., 2004, human Roberts-Thomson et al., 2009, horse Van Loon et al., 2002). Middle row, right panel: atrial CV as a function of body mass [mouse Leaf et al., 2008, rat Haugan et al., 2005, rabbit de Groot et al., 2003, dog Fareh et al., 1998, goat Verheule et al., 2010, human Hansson et al., 1998, horse (NA)]. Bottom row, left panel: wavelength (product of CV and ERP) as a function of body mass. Bottom row, right panel: ratio of atrial circumference to wavelength. The atrial circumference was calculated by taking the LA diameter and approximating the total circumference as 2^*^2^*^π^*^LA radius (mouse Blana et al., 2010, rat Boixel et al., 2003, rabbit Hirose et al., 2005, dog Stepien et al., 1998, goat Wijffels et al., 1997, human Monnig et al., 2005, horse Menzies-Gow et al., 2005). Interestingly, the number of reentrant waves that can fit on the atrial circumference does not scale strongly with the body mass.

The simplest form of reentry is a single reentrant wave circling around an anatomic obstacle or area of functional block. The “wavelength” of such a wave is the product of CV and ERP. (Wiener and Rosenblueth, 1946) With the normal CV and ERP in the mouse heart, this wavelength (WL) would be approximately 1.2 cm in for the atrium, close to the normal estimated atrial circumference (Figure 2). However, this same argument holds true for larger species, as the ratio between estimated atrial circumference to WL is almost completely independent of body mass (Figure 2). According to the WL theory, a decrease in CV or ERP would make reentry more likely. Indeed, shortening the ERP by applying acetylcholine increases AF stability in mice, as it does in larger species (Kovoor et al., 2001; Wakimoto et al., 2001).

A shorter WL would also allow more fibrillation waves to propagate simultaneously within the atria or ventricles. According to the “multiple wavelet theory,” fibrillation can persist without any particular site or circuit dominating the arrhythmia, as long as a sufficiently large number of irregularly propagating fibrillation waves can coexist within a substrate (Allessie, 1998). The likelihood of multiple wavelet reentry increases when WL becomes smaller (Rensma et al., 1988; Allessie et al., 1996). However, several studies have shown that structural inhomogeneities can lead to “zig-zag” conduction, allowing “microreentry” to occur in much smaller circuits than would be expected based on calculation of the macroscopically determined WL (de Bakker et al., 1993; Spach and Josephson, 1994; Koura et al., 2002). To date, there is no direct evidence that “anarchical” multiple wavelet reentry can occur in mouse hearts. The small substrate size represented by the mouse heart may limit the number of coexisting fibrillation waves and thus make multiple wavelet reentry less likely. For example, the degree of fibrosis necessary to create a substrate for multiple wavelet reentry in a mouse heart may have to be larger than that required in a larger heart. AF stability was indeed greatly increased in a mouse model of selective atrial fibrosis (Verheule et al., 2004), but fibrillatory conduction in this model may be based on triggered activity rather than multiple wavelet reentry (Choi et al., 2012).

Spiral wave reentry is a specific form of reentry that has been described mathematically and observed experimentally in several types of excitable media (Jalife et al., 2002; Comtois et al., 2005). Vaidya et al. demonstrated sustained spiral wave reentry around a single core in murine ventricles with a period of wavefront rotation of 72 ms and frequency of 14 Hz (Vaidya et al., 1999). This study also detected figure-of-8 reentry with a rotation period of 66 ms, demonstrating that the murine ventricle is able to sustain two stable reentrant waves (Vaidya et al., 1999). It is important to note that calculated wavelengths in this study were much larger than size of the mouse heart and that APD showed almost no frequency dependence (Vaidya et al., 1999). Computer simulations suggested that the core during vortex reentry altered repolarization in adjacent myocardium, leading to reduced APDs near the core and longer APDs at larger distance to the core (Beaumont et al., 1998). This hypothesis was supported by the observation that during occurrence of spiral wave reentry, the average APD of the entire preparation was reduced and that pacing at the frequency of the spiral wave produced longer APDs (Vaidya et al., 1999). Thus, wavelength calculations based on external electrical stimulation probably have a poor predictive value for generation and maintenance of spiral wave reentry in the murine heart (Vaidya et al., 1999).

Studies in mammalian species from mouse to horse show scaling of VF frequency with BM, following the equation VF_frequency_ = 18.9 × BM^−1/4^ (Noujaim et al., 2007). Noujaim et al. demonstrated stable VF with vortex-like reentry and highest dominant frequency (DF) in the mouse heart with 38 Hz and reduction of DF with growing BM, as reflected by a DF of 6.8 Hz in the human heart (Noujaim et al., 2007). However, in larger mammalian hearts, the rapid activity of a rotor in one region does not conduct in a 1:1 fashion to the rest of the substrate. In most cases, and certainly within the complex anatomy of the atria, the waves emanating from that “mother” rotor will break up and spin off “daughter” wavelets to the rest of the atrium (Chen et al., 2000). Thus, the overall conduction pattern will be characterized by interaction of the rotor and fibrillatory waves in the rest of the substrate. When a rotor is present in a relatively small medium, the interaction between the rotor and the rest of the substrate is therefore likely to be different (Zou et al., 2005).

### Ectopic activity

Cellular proarrhythmic events can give rise to ectopic activity. Accelerated diastolic depolarization of myocytes with pacemaker activity can cause a latent pacemaker region to act as an ectopic focus (enhanced automaticity). By contrast, triggered activity results from “afterdepolarizations” triggered by a normal action potential. Early afterdepolarizations (EADs) develop during phase 2–3 of the AP and are precipitated by a prolonged APD during which the L-type Ca^2+^ channels recover and switch from the inactivated to the open state to produce a depolarizing current (Sabir et al., 2008). Delayed afterdepolarizations (DADs) occur during phase 4 of the AP in situations of intracellular Ca^2+^ overload, evoked by e.g., beta-adrenergic stimulation, ischemia, and hypokalaemia. In this case, the excess of intracellular Ca^2+^ is transported by the NCX, causing a depolarizing current (Schotten et al., 2011). As discussed in section III, to function at high heart rates, mouse myocytes have a short action potential and some differences in calcium handling compared to myocytes from larger mammalian hearts, and these factors may affect the generation of EADs and DADs. Nevertheless, several mouse models have shown arrhythmias initiated by afterdepolarizations (see e.g., Fabritz et al., 2003; Killeen et al., 2007a; Choi et al., 2012; Li et al., 2012; Pott et al., 2012).

Regardless of its exact nature (diastolic depolarization, early or delayed after depolarization), a proarrhythmic mechanism active in a single myocyte is unlikely to lead to a propagated response in the intact tissue, because the current generated by that myocyte will leak away to its neighbors without reaching the action potential threshold. Thus, the occurrence of a proarrhythmic event will have to be synchronized in a sufficiently large group of myocytes in order to reach the threshold and be able to propagate away from that area. This holds true both for an ectopic pacemaker region (Joyner et al., 2000) and for an area displaying triggered activity (Sato et al., 2009). Does an ectopic focus in a mouse heart have to same relative size to the rest of the heart as a focus in larger mammals? Mouse ventricular myocytes (90 μm in length, 14 μm in diameter) (Toischer et al., 2010) are smaller than dog myocytes (130 μm in length, 30 μm in diameter) (Clemo et al., 1998). Assuming myocytes to be roughly cylindrical, this would translate to a ratio of 8:1 in myocyte volume between dogs and mice. While considerable, this ratio is much smaller than the 1100:1 ratio in ventricular weight (or volume) between these species (Bienvenu and Drolet, 1991), implying that the mouse ventricle contains far fewer myocytes (by about a factor 150 in this crude estimate). No direct measurements of electrotonic interactions in mouse ventricles are available, but at 0.6 m/s, the conduction velocity in mouse ventricles is close to that in larger species, suggesting that electrotonic interactions are comparable (Gutstein et al., 2001). These indirect arguments would indicate that an ectopic focus in mouse ventricles may not be much smaller than that in larger hearts, and would thus occupy a relatively larger area of the heart. During rapid focal activity, this size-relation would affect the pattern of fibrillation (i.e., wavebreak). In addition, the small size of the mouse heart would limit the number of localized arrhythmogenic sources that can coexist. Propagation from an ectopic focus is most likely when electrical coupling gradually increases from the focus to the surrounding muscle, because a high degree of electrical coupling would effectively silence the focus (Joyner et al., 2007). Thus, structural remodeling (e.g., fibrosis) would allow a smaller focus to successfully conduct its rapid rate to the rest of the heart.

Apart from the role of cardiac size, various other caveats apply to extrapolation of arrhythmias in mouse models to clinically observed human arrhythmias. A very high degree of overexpression or underexpression of a particular gene may lead to indirect alterations that would not be observed as a result of more moderately altered expression of that gene in human pathology. In addition, overexpression or deletion of a gene during the prenatal period may lead to a disruption of cardiac development and result in secondary changes that do not reflect the role of that gene in the adult human heart. All cautionary considerations notwithstanding, mouse models have displayed numerous types of arrhythmias that bear a striking electrocardiographic resemblance to human arrhythmias. These include various tachyarrhythmias such as atrial flutter (Zhang et al., 2005), atrial fibrillation (Verheule et al., 2004), (polymorphic) ventricular tachycardia (Fabritz et al., 2003; van Rijen et al., 2004; Cerrone et al., 2007) and ventricular fibrillation (Cerrone et al., 2005), bradyarrhythmias such as sinus bradycardia (Lei et al., 2005) and conduction disorders such as AV block (Zhang et al., 2011), bundle branch block (van Rijen et al., 2001), accessory atrioventricular pathways (Arad et al., 2003) and long QT (Salama and London, 2007; Ruan et al., 2009; Sanguinetti, 2010) and Brugada (Martin et al., 2010) syndromes.

## Conclusions

Over the last two decades, mice have become a widely used model instrument in research into cardiac arrhythmias. Progress in miniaturization of techniques has allowed detailed studies of electrophysiological parameters of the murine heart. Genetic engineering has allowed the generation of mouse models carrying human mutations that cause arrhythmias and has contributed to understanding the role of specific genes, proteins and ionic channels in arrhythmogenesis. In general, mouse models have broadened our knowledge of cardiac electrophysiology and mechanisms underlying atrial and ventricular arrhythmias in humans. Mouse models can mimic human cardiac arrhythmias despite differences in cardiac electrophysiology. However, some mechanisms of arrhythmias in mice may differ from those in humans and therefore have to be extrapolated to the human situation with caution.

### Conflict of interest statement

The authors declare that the research was conducted in the absence of any commercial or financial relationships that could be construed as a potential conflict of interest.
